# Association between *Chlamydia trachomatis*, *Neisseria gonorrhea*, *Mycoplasma genitalium*, and *Trichomonas vaginalis* and Secondary Infertility in Cameroon: A case-control study

**DOI:** 10.1371/journal.pone.0263186

**Published:** 2022-02-04

**Authors:** Clarisse Engowei Mbah, Amy Jasani, Kristal J. Aaron, Jane-Francis Akoachere, Alan T. N. Tita, William M. Geisler, Barbara Van Der Pol, Jodie Dionne-Odom, Jules Clement Assob Ngeudia

**Affiliations:** 1 Department of Microbiology and Parasitology, University of Buea, Buea, Cameroon; 2 Institute of Medical Research and Medicinal Plants Studies, Center for Research on Health and Priority Pathologies, Yaoundé, Cameroon; 3 Department of Medicine, Division of Infectious Diseases, University of Alabama at Birmingham, Birmingham, AL, United States of America; 4 Department of Obstetrics and Gynecology, Division of Maternal Fetal Medicine, University of Alabama at Birmingham, Birmingham, AL, United States of America; 5 Department of Laboratory Medicines, Faculty of Medicine and Pharmaceutical Sciences, University of Douala, Douala, Cameroon; University of Texas Health Science Center at San Antonio, UNITED STATES

## Abstract

**Objective:**

Data on the prevalence and etiology of infertility in Africa are limited. Secondary infertility is particularly common, defined as the inability of a woman to conceive for at least one year following a full-term pregnancy. We describe a prospective study conducted in Cameroon designed to test the hypothesis of an association between common treatable sexually transmitted infections (STI): *Chlamydia trachomatis* (CT), *Neisseria gonorrhoeae* (NG), *Mycoplasma genitalium* (MG), and *Trichomonas vaginalis* (TV) and secondary infertility in women.

**Methods:**

In this case-control study, we enrolled women in Fako Division, Cameroon between November 2017 and December 2018 with secondary infertility (cases) or current pregnancy (controls). We conducted a baseline survey to collect sociodemographic, and sexual and medical history information. Nucleic acid amplification testing using Aptima (Hologic, San Diego, CA, US) was performed on endocervical swabs for CT, NG, MG, and TV. Multivariable logistic regression was used to assess the relationship between active STI and secondary infertility.

**Results:**

A total of 416 women were enrolled: 151 cases and 265 controls. Compared to controls, cases were older (median age 32 vs 27 years) and had more lifetime sexual partners (median 4 vs 3) (p<0.001). Cases were more likely to report dyspareunia, abnormal menses, prior miscarriage, and ectopic pregnancy (all p<0.05). STI positivity was not significantly different among cases and controls (2.7% vs 5.4% for CT, 1.3% vs 2.9% for NG, 6.0% vs 7.0% for MG, respectively), with the exception of TV which was more common in pregnant controls (0.7% vs 5%; p = 0.02).

**Conclusion:**

Study findings did not support an association between active STI and secondary infertility in Cameroon. Given high rates of pre-existing tubal damage, routine STI screening and treatment in younger women may be more impactful than costly STI testing during infertility assessments.

## Introduction

Infertility affects 10–15% of couples, an estimated 80 million people globally [1]. Secondary infertility is defined as the inability to conceive for at least one year by a woman with a history of prior delivery. In Cameroon, a country in central Sub-Saharan Africa (SSA), the prevalence of infertility ranges from 15% to 30% and secondary infertility is twice as common as primary infertility [2, 3]. Stigma associated with infertility is common in SSA and women in couples with infertility are more likely to experience domestic violence, divorce, and social marginalization compared to women without infertility [2, 4]. Additional studies are needed to understand the etiology of infertility in SSA to inform prevention and treatment options in a region where access to assisted reproductive technology is limited [4, 5].

*Chlamydia trachomatis* (CT) and *Neisseria gonorrhoeae* (NG) infections are common bacterial sexually transmitted infections (STI) that cause cervicitis in women and ascend to the upper reproductive tract in 10–20% of cases, leading to pelvic inflammatory disease (PID). PID can cause inflammation and damage to fallopian tubes that results in tubal factor infertility (TFI) [6, 7]. CT infection is associated with a 3.4-fold increase in TFI [8]. Data on the association between *Mycoplasma genitalium* (MG) infection and infertility in women are limited and *Trichomonas vaginalis* (TV) infection is not known to impact fertility. Studies of MG, TV and infertility using highly sensitive PCR molecular diagnostic testing are ongoing but this STI testing is mostly unavailable in SSA [9, 10]. Since women with secondary infertility have a prior history of delivery, they may be more likely to have a reversible cause of TFI, such as a recently acquired STI. If active infection is present, it is possible that STI treatment could reduce tubal inflammation and improve fertility. This case-control study was designed to test our hypothesis of an association between active common STIs and secondary infertility among women in Cameroon.

## Methods

### Study area and setting

The study was conducted in semi-urban areas of Fako Division (Buea, Limbe Tiko, and Mutengene) in the Southwest region of Cameroon in Central Africa. Participants were seen at the main reference hospital in the region. Cases with secondary infertility were enrolled at the gynecological unit and pregnant controls were enrolled at antenatal clinics in the same region.

### Study design and participants

This unmatched case-control study enrolled women in the age range of 15–46 years. Cases were women with secondary infertility according to self-report or as identified by a gynecologist. Secondary infertility was defined as the inability to conceive by a woman despite at least one year of unprotected sexual intercourse following a prior delivery. Controls were randomly selected pregnant women seen in prenatal clinic who reported no history of infertility. Approximately two controls were selected for each case identified. Women were ineligible to participate if they refused to grant consent and if they had taken any antibiotics during the past 7 days.

### Study procedures

Women were enrolled in the study after providing informed consent. Gynecologists, nurses, and study staff surveyed women about socio-demographics, sexual history, obstetrical and gynecological history, including any infertility evaluations. Medical records were reviewed for STI history, pregnancy history, and other medical history, when available. Questionnaire and study procedure was formulated to follow hospital patient management protocols at the various study sites.

### Biological specimen collection

Endocervical swabs were collected by a medical provider using Dacron swabs and placed directly into Hologic^®^ Aptima^®^ vaginal swab transport media (Hologic Inc San Diego, USA). Specimens were transported to the University of Buea Laboratory and stored at -80°C until transport to the University of Alabama at Birmingham STI Laboratory (USA) for STI nucleic acid amplification testing (NAAT).

### Laboratory procedures

Three Aptima (Hologic, San Diego, CA) assays were performed from a single endocervical specimen on the Panther (Hologic San Diego, CA) platform: Aptima Combo 2 (AC2) for CT and NG [11, 12], Aptima TV (ATV) [13], and Aptima MG (AMG) [14].

### Ethical considerations

The study was approved by the Faculty of Health Sciences Institutional Review Board in Cameroon (No 2016/459/UB/FHS/IRB). Administrative clearance was also obtained from the Regional Delegation of Public Health for the Southwest Region and all the health facilities where study was conducted.

### Statistical analysis

For a power calculation assuming two controls per case, a sample size of 408 (136 cases and 272 controls) would provide >80% power at an alpha level of 0.05 to detect an association between STI and secondary infertility if STI positivity in the control group was 19%.Chi-square or Fisher’s exact testing was used for comparisons of categorical variables where appropriate; and, analysis of variance or the Kruskal-Wallis test was used for continuous variables. Univariate logistic regression was performed to identify predictors of secondary infertility. Variables of interest, specifically socio-demographics, sexual behaviors, and a composite STI measure were included in the full multivariable logistic regression based on published data to explore the association between STI positivity and secondary infertility. Unadjusted and adjusted ORs, 95% CI, and p-values were calculated with significance set at p<0.05. SAS version 9.4 (Cary, NC) was used for analyses.

## Results

### Participant characteristics

A total of 416 women were enrolled: 151 cases and 265 controls (Table 1). Cases were older than controls, median (interquartile range) 32 years (27, 36) vs 27 years (24, 32), p<0.001. Sexual behavior differed somewhat between the groups: 26% of cases and 7% of controls had ≥5 lifetime partners (p<0.001) although the median number of lifetime partners was <5 in both groups. Two-thirds of women in both groups were married and cases were more likely to report multiple current sexual partners (4% cases vs 0.8% controls; p = 0.03).

**Table 1:** Participant characteristics for women with secondary infertility (cases) and pregnant women (controls).

Variables	Total n = 416	Cases n = 151	Controls N = 265	p-value
	n	n	n	
**Sociodemographic Data**				
**Age, years**				
**15–24**	95 (22.9%)	14 (9.23%)	81 (30.7%)	<0.001
**25–29**	130 (31.3%)	43 (28.5%)	87 (33.0%)	
**30–39**	127 (30.6%)	54 (35.8%)	73 (27.7%)	
**40–46**	64 (154%)	40 (26.5%)	24 (9.1%)	
**Occupation**				<0.001
**White Collar**	106 (25.5%)	52 (34.4%)	54 (20.4%)	
**Business**	187 (45.0%)	70 (46.4%)	117 (44.2%)	
**None**	77 (18.5%)	17 (11.3%)	60 (22.6%)	
**Student**	46 (11.1%)	12 (8.0%)	34 (12.8%)	
**Marital Status**				0.907
**Married**	274 (65.9%)	100 (66.2%)	174 (65.7%)	
**Single, divorced, or widowed**	142 (34.1%)	51 (33.8%)	91 (34.3%)	
**Education**				0.385
**None or primary**	76 (18.3%)	26 (17.2%)	50 (18.9%)	
**Secondary, High School, or Vocational**	235 (56.5%)	81 (53.6%)	154 (58.1%)	
**University**	105 (25.2%)	44 (29.1%)	61 (23.0%)	
**Sexual History**				
**Age of sexual debut, years**				0.876
**<14**	14 (3.7%)	6 (4%)	8 (3.5%)	
**14–19**	282 (74.4%)	113 (75.3%)	169 (73.8%)	
**≥20**	83 (21.9%)	31 (20.7%)	52 (22.7%)	
**Lifetime sex partners**				<0.001
**1–5**	313 (85.5%)	110 (74.3%)	203 (93.1%)	
**≥5**	53 (14.5%)	38 (25.7%)	15 (6.9%)	
**Currently more than one sexual partner**	8 (1.9%)	6 (4.0%)	2 (0.8%)	0.029
**Persistent Dyspareunia**	132 (35.9%)	81 (54.4%)	51 (23.3%)	<0.001
**OB/Gyn History**				
**Last menstrual period was abnormal**	40 (9.6%)	28 (18.5%)	12 (4.5%)	<0.001
Menstrual cycle duration, days*				0.331
**21–26**	76 (18.3%)	22 (14.6%)	54 (20.4%)	
**27–29**	211 (50.7%)	78 (51.7%)	133 (50.2%)	
**30–35**	39 (9.4%)	16 (10.6%)	23 (8.7%)	
**>35**	6 (1.4%)	4 (2.7%)	2 (0.8%)	
**Age of menarche, years**				0.342
**8–10**	3 (0.83%)	2 (1.4%)	1 (0.5%)	
**11–13**	95 (26.3%)	40 (28.8%)	55 (24.8%)	
**14–16**	236 (65.4%)	90 (64.8%)	146 (65.8%)	
**17–26**	27 (7.5%)	7 (5.0%)	20 (9.0%)	
**Result of test for ovulation**				0.100
**Regular**	41 (67.2%)	20 (57.1%)	21 (80.8%)	
**Irregular**	17 (27.9%)	12 (34.3%)	5 (19.2%)	
**Absence of ovulation**	3 (4.9%)	3 (8.6%)	0 (0%)	
**History of miscarriage**	83 (20.0%)	44 (29.1%)	39 (14.7%)	<0.001
**History of stillbirth**	14 (3.4%)	3 (2.0%)	11 (4.2%)	0.239
**History of ectopic pregnancy**	9 (2.2%)	7 (4.6%)	2 (0.8%)	0.013
**Number of prior pregnancies**				<0.001
**1**	171 (41.1%)	76 (50.3%)	95 (35.9%)	
**2**	133 (32.0%)	68 (45.0%)	65 (24.5%)	
**≥3**	112 (26.9%)	7 (4.6%)	105 (39.6%)	
**Duration of infertility (years)**				<0.001
**0**	265 (63.7%)	0 (0%)	265 (100%)	
**1–5**	106 (25.5%)	106 (70.2%)	0 (0%)	
**>5**	45 (10.8%)	45 (29.8%)	0 (0%)	
**Sexually Transmitted Infections**				
**CT Positive NAAT**	17 (4.3%)	4 (2.7%)	13 (5.4%)	0.198
**NG Positive NAAT**	9 (2.3%)	2 (1.3%)	7 (2.9%)	0.492
**MG Positive NAAT**	26 (6.6%)	9 (6%)	17 (7.1%)	0.676
**TV Positive NAAT**	13 (3.3%)	1 (0.7%)	12 (5.0%)	0.020
**STI coinfection (>1 NAAT+)**	5 (1.5%)	1 (0.7%)	4 (2.0%)	0.652
**Any STI (CT/NG/MG/TV)**	59 (14.2%)	15 (10.0%)	44 (18.2%)	0.028

Note: Parametric p-values were calculated by chi-square test while non-parametric p-values were calculated by Fisher’s exact test.

*Data were missing for >10% of individuals in both cases and controls.

Most cases (95%) had 1–2 prior pregnancies and 40% of controls had ≥3 prior pregnancies (p<0.01). Majority of the cases (62.3%) reported to have consulted before for infertility. The duration of infertility was prolonged (>5 years) in 30% of cases. Persistent dyspareunia (painful sex) was reported by 54% of cases and 23% of controls (p<0.001). Women with secondary infertility were more likely to report abnormal periods (19% vs 5%), prior miscarriage (29% vs 15%) and ectopic pregnancy (5% vs 1%) compared to pregnant controls (all p<0.05).

### STI prevalence and tubal patency

**Fig 1:**
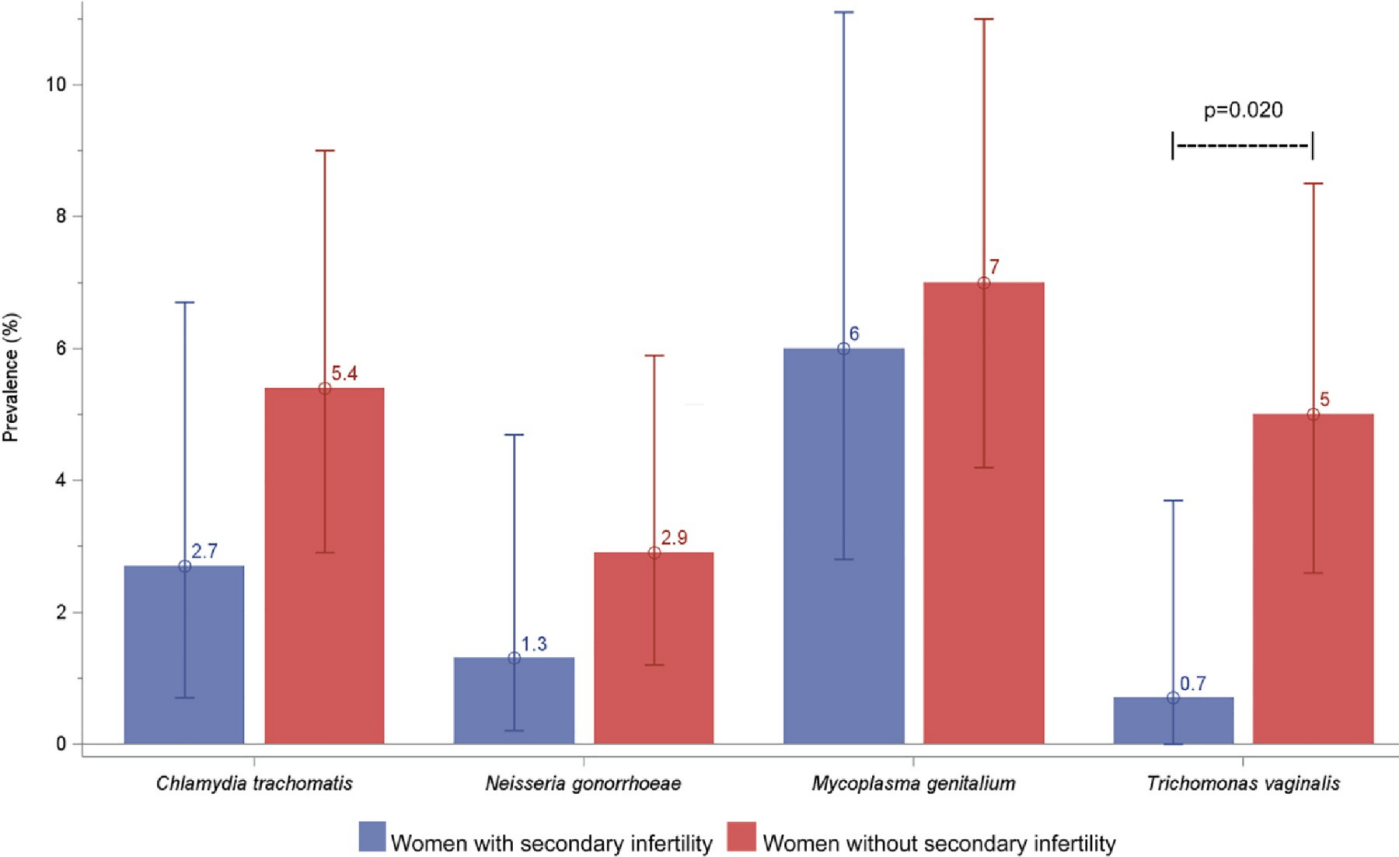
STI prevalence (CT/NG/MG/TV) among cases and controls.

Positivity rates for CT, NG, and MG ranged from 1.3–7.0% and were not significantly different between cases and controls (2.7% vs 5.4% for CT, 1.3% vs 2.9% for NG, and 6.0% vs 7.0% for MG, respectively). TV positivity was higher in controls (0.7% vs 5.0%; p = 0.02) (Fig 1). The composite STI measure (CT/NG/MG/TV) was 10.0% in cases and 18.2% in controls (p = 0.03). STI coinfections were infrequently detected in 0.7% of cases and 2% of controls.

Among cases, 46 had hysterosalpingography (HSG) results in their medical record. Of these 46, 31 women (67%) had documented evidence of tubal obstruction +/- hydrosalpinx (20 with bilateral obstruction, 11 with unilateral obstruction), 6 had uterine adhesions, and 1 had radiographic evidence of cervicitis. The STI positivity (CT/NG/MG/TV) amongst cases with HSG was 3/46 (6.5%). None of the controls had HSG results as a comparison group since they had no prior evidence of infertility according to inclusion criteria.

### Factors associated with secondary infertility

In unadjusted models with secondary infertility as the outcome, older age, white collar occupation, higher number of lifetime sex partners and having more than one current sex partner were associated with infertility (Table 2). When compared to the youngest age category of 15–24 years, the crude odds ratios (OR) and 95% confidence intervals for secondary infertility increased in a stepwise fashion from 2.9 (CI 1.5–5.6) for women aged 25–29 years to 9.6 (95% CI 4.5–20.6) for women aged 40–46 years. Women with more than one current sex partner (OR 5.4; 95% CI 1.1–27.3) and ≥5 lifetime sex partners (OR 4.7; 95% CI 2.5–8.9) had greater odds of secondary infertility. Women with dyspareunia, abnormal periods, history of miscarriage and prior ectopic pregnancy also had greater odds of secondary infertility (Table 2).

**Table 2:** Factors associated with secondary infertility in cameroon: Unadjusted and adjusted models.

Variable	Category	Unadjusted Odds Ratio (95% CI)	p-value	Adjusted Odds Ratio (95% CI)	p-value
**Age, years**	15–24	-	-	-	-
	25–29	2.86 (1.46–5.61)	0.002	2.28 (1.10–4.74)	0.028
	30–39	4.28 (2.20–8.34)	<0.001	3.32 (1.56–7.06)	0.002
	40–46	9.64 (4.51–20.6)	<0.001	5.62 (2.39–13.2)	<0.001
**Occupation**	White Collar	2.73 (1.28–5.84)	0.01	1.42 (0.58–3.44)	0.441
	Business	1.70 (0.82–3.49)	0.15	1.23 (0.51–2.96)	0.647
	None	0.80 (0.34–1.88)	0.61	0.64 (0.24–1.72)	0.374
	Student	-	-	-	-
**Marital Status**	Married	0.98 (0.64–1.49)	0.91		
	Single, divorced, or widowed	-	-		
**Education**	None or primary	0.72 (0.39–1.33)	0.30	0.85 (0.38–1.94)	0.707
	Secondary, High School, or Vocational	0.73 (0.45–1.17)	0.19	0.73 (0.41–1.32)	0.302
	University	-	-	-	-
**Age of sexual debut**	<14 years	1.26 (0.40–3.97)	0.70		
	14–19 years	1.12 (0.68–1.86)	0.66		
	≥20 years	-	-		
**Lifetime sex partners**	1–5	-	-	-	-
	≥5	4.67 (2.46–8.88)		4.02 (1.97–8.21)	<0.001
**More than one partner**	No	-	-	-	-
	Yes	5.44 (1.08–27.3)	0.04	2.36 (0.38–14.6)	0.357
**Dyspareunia**	Always or sometimes	3.92 (2.50–6.15)	<0.001		
	Never	-	-		
**Last menstrual period was normal**	No	4.80 (2.36–9.75)	<0.001		
	Yes	-	-		
**Age of menarche category**	8–10 years	5.71 (0.45–73.1)	0.18		
	11–13 years	2.08 (0.80–5.38)	0.13		
	14–16 years	1.76 (0.72–4.33)	0.22		
	17–26 years	-	-		
**History of miscarriage**	No	-	-		
	Yes	2.38 (1.46–3.88)	<0.001		
**History of stillbirth**	No	-	-		
	Yes	0.47 (0.13–1.70)	0.25		
**History of ectopic pregnancy**	No	-	-		
	Yes	6.39 (1.31–31.2)	0.02		
**Number of prior pregnancies**	1	-	-		
	2	1.31 (0.83–2.06)	0.25		
	≥3	0.08 (0.04–0.19)	< .001		
**CT NAAT**	Negative	-	-		
	Positive	0.48 (0.15–1.50)	0.21		
**NG NAAT**	Negative	-	-		
	Positive	0.45 (0.09–2.20)	0.33		
**MG NAAT**	Negative	-	-		
	Positive	0.84 (0.36–1.94)	0.69		
**TV NAAT**	Negative	-	-		
	Positive	0.13 (0.02–0.99)	0.05		
**Any STI (CT, NG, MG, TV)**	No	-	-	-	-
	Yes	0.50 (0.27–0.93)	0.03	0.73 (0.36–1.51)	0.400

The multivariable model included 348 women and adjusted for age, educational level, occupation, number of lifetime sex partners, number of current sex partners, and any STI (CT, NG, MG, TV) (Table 2). Older age remained statistically significant in a stepwise fashion: the adjusted odds ratios (aOR) for secondary infertility was 2.3 (95% CI 1.1–4.7) for age 25–29 years, aOR 3.3 (95% CI 1.6–7.1) for age 30–39 years, and aOR 5.6 (95% CI 2.4–13.2) for age 40–46 years, all compared to age 15–24 years. Also, women with ≥5 lifetime sex partners had greater odds of secondary infertility (aOR 4.0 [95% CI 2.0–8.2]). STI positivity was not associated with infertility (Table 2).

## Discussion

This is one of the first studies to focus on the association between active STI and secondary infertility in Africa using highly sensitive molecular diagnostic testing to detect CT, NG, MG, and TV in women of reproductive age in Cameroon. Our prospective case-control study did not support our hypothesis of a possible association between active STI and secondary infertility although classic STI risk factors (higher number of lifetime sexual partners, more than one current sexual partner) were more prevalent in cases compared to controls.

STI prevalence among women in this study was similar to other studies in Africa [15–20]. Among the four STIs detected, only TV showed a significant difference between the two groups, with higher positivity in controls. Additional studies are needed to explore the association noted in unadjusted models between TV and pregnancy. Any assessment of the association between prior STI infection and secondary infertility in Cameroon is limited by the fact that, unlike high-income countries, routine STI testing among women is not recommended and sensitive diagnostic testing is mostly unavailable. The performance of syndromic STI management in SSA as recommended by the World Health Organization is demonstrably poor, particularly in women who normally have asymptomatic bacterial STI [21].

In terms of STI and TFI, tubal blockage has been detected in 50% of women with infertility (mostly secondary infertility) in urban Cameroon [22]. This is consistent but lower than the 67% of women with secondary infertility shown to have tubal blockage in the semi urban setting of this present study. The mechanism of pathogenesis of NG and CT fallopian tube infection and inflammation resulting in tubal infertility has been well-described [23]. In the literature, tubal obstruction is seen in 20–30% of cases of primary and secondary infertility [24]. Egbe *et al*. found that a history of CT was strongly associated with tubal factor infertility in Cameroon [25]. A meta-analysis demonstrated a higher prevalence of NG in infertile populations compared to the general population [16]. Although data on MG are fewer, a recent US study by Peipert *et al* found an association between serologic evidence of prior MG infection and infertility [26, 27].

Our study did not show an association between active infection with CT, NG, MG, and secondary infertility. These findings do not rule out a possible association between bacterial STI and secondary infertility since HSG test results among participants with secondary infertility suggested that tubal damage had already occurred. One relevant implication of our study is that current infertility management protocols in Cameroon that rely on CT serology testing and antibiotic treatment, may be ineffective and costly for the patient if infection occurred in the past. Therefore, the WHO recommended syndromic STI management and treatment (which fails to detect asymptomatic infections) may not be an effective strategy to prevent tubal damage and adverse pregnancy outcomes. More frequent and earlier STI screening and treatment of women in SSA may be needed to prevent upper genital tract complications [28]. In addition to highly sensitive molecular diagnostic testing, it would be useful to measure STI-specific host responses to detect prior infection and further understand the role of each STI in terms of a causal pathway for TFI. A serologic study to compare immunoglobulin-specific responses to CT between cases and controls in our population will be informative about prior CT exposure and is currently ongoing. We have previously shown that CT antibody testing such as IgG1 to elementary bodies and CT OmcB can help elucidate the timing of prior infection [29].

Some factors associated with secondary infertility in our study align with other studies; age, adverse pregnancy outcomes (miscarriage and ectopic pregnancy) and dyspareunia [30, 31]. Modifiable risk factors such as marital status, education, and age of sexual debut had no association with secondary infertility in our study. This differs from findings by Larsen *et al*. in Cameroon where secondary infertility was associated with early sexual debut (before the age of 15) and single marital status [32].

Our study had limitations. Importantly, cases and controls were not age-matched, duration of secondary infertility varied from 1-18years among cases and there was heterogeneity in lifetime number of sexual partners between groups (although a majority reported 1–5 partners). Since the risk of STI generally decreases with age and cases were older than pregnant controls, this may have biased our sample toward higher STI rates in pregnant controls (not observed for CT/NG/MG). Participant recall error and bias in STI history information are also possible since STI testing can be performed as part of the initial infertility evaluation. If cases received empiric STI treatment during infertility evaluation, they may have had more historical antibiotic exposure compared to controls. Finally, since the study sample was isolated to women in Southwest Cameroon, findings may not generalize to all women with secondary infertility.

Future studies should include detailed diagnostic evaluations to better understand the most common etiologies of primary and secondary infertility among women and men in sub-Saharan Africa as well as the frequency and etiology of tubal damage. Once the population attributable fraction due to STI is understood, prospective prevention studies can be designed.

## Conclusion

This prospective case-control study of women with secondary infertility in Cameroon did not show an association with active STI (CT, NG, MG, or TV). Since rates of tubal damage were high, the benefit of routine CT/GC NAAT testing and prompt treatment in young sexually active women for the endpoint of infertility prevention should be investigated further.

## Supporting information

S1 Dataset (XLSX)
